# 

**Published:** 1859-12

**Authors:** 


### DENTAL REGISTER

### OF THE WEST.

EDITED BY

J. TAFT AND GEO. WATT.

December, 1859

MONTHLY.--THREE DOLLARS PER ANNUM, IN ADVANCE.

CINCINNATI:
J. T. TOLAND, PUBLISHER AND PROPRIETOR.
1859.

